# Supplementary material for: High-throughput screening for discovery of benchtop separations systems for selected rare earth elements
Source: Commun Chem. 2020 Jan 20;3:7. doi: 10.1038/s42004-019-0253-x (PMC9814905; doi:10.1038/s42004-019-0253-x)
Supplement: Supplementary file 2 — Supplementary Information [file 42004_2019_253_MOESM2_ESM.pdf]

Supplementary Information

High-Throughput Screening for Discovery of Benchtop Separations Systems for Selected Rare Earth Elements

Joshua J. M. Nelson[1], Thibault Cheisson[1], Haley J. Rugh[1], Michael R. Gau[1], Patrick J. Carroll[1], Eric J. Schelter[1]*

[1]P. Roy and Diana T. Vagelos Laboratories, Department of Chemistry, University of Pennsylvania, 231 S 34th Street, Philadelphia, Pennsylvania 19104

*E-mail: schelter@sas.upenn.edu

Supplementary Methods 2
General Methods 2
Materials 2
Inductively Coupled Plasma Optical Emission Spectroscopy Measurements 2
X-Ray Crystallography 3
High Throughput Precipitation Screening 4
General Procedure for Separation of Rare Earth Mixtures 5
Procedure for Ligand Recovery 5
Synthetic Details and Characterization 6
2-chloropyridine-3-carboxylic acid *N*-oxide (**B**) 6
1-hydroxy-2-oxo-1,2-dihydropyridine-3-carboxylic acid (**C**) 6
H_3tren-1,2,3-HOPO (H_3**1**·TFA) 7
La(tren-1,2,3-HOPO) (**1·La**) 7
Nd(tren-1,2,3-HOPO) (**1·Nd**) 8
Dy(tren-1,2,3-HOPO) (**1·Dy**) 8
NMR Spectra 9
FT-IR Spectra 15
Supplementary References 16

Supplementary Methods

General Methods. Reactions were performed under ambient conditions unless otherwise specified. Reactions performed under inert atmosphere were performed using standard Schlenk techniques or in a drybox equipped with a molecular sieves 13X / Q5 Cu-0226S catalyst purifier system. Related glassware was oven-dried for at least 3 hours at 150 °C prior to use. ^{1}H and ^{13}C{^{1}H} NMR spectra were obtained on a Bruker DMX-300 Fourier transform NMR spectrometer at 300 MHz and a Bruker AVIII-400 NMR spectrometer at 100.61 MHz, respectively, or on a Bruker DRX-500 NMR spectrometer at 500 MHz and 126 MHz, respectively. Accurate mass measurement analyses were conducted on either a Waters GCT Premier, time-of-flight, GCMS with electron ionization (EI), or an LCT Premier XE, time-of-flight, LCMS with electrospray ionization (ESI). Samples were taken up in a suitable solvent for analysis. The signals were mass measured against an internal lock mass reference of perfluorotributylamine (PFTBA) for EI-GCMS, and leucine enkephalin for ESI-LCMS. Waters software calibrates the instruments, and reports measurements, by use of neutral atomic masses. The mass of the electron is not included. All dilutions were performed using appropriate class A volumetric glassware. Elemental analyses were performed on a Costech ECS 4010 analyzer. Fourier transform-infrared (FT-IR) measurements were collected on a Jasco FT/IR-480 Plus spectrometer of samples as KBr pellets.

Materials. All materials were purchased directly from commercial sources and used without further purification, unless otherwise noted. Anhydrous tetrahydrofuran (THF) was sparged for 20 minutes with dry argon and dried using a commercial two-column solvent purification system comprising columns packed with neutral alumina. MilliQ water used was obtained from a Millipore reverse osmosis purification system and filtered through a 0.2 micron filter prior to use. ~1M NaOH solutions were prepared by dissolving solid NaOH in MilliQ water. The exact concentration was determined by titrating against a measured quantity of potassium hydrogen phthalate (KHP) dissolved in water with 2 drops of phenolphthalein indicator; performed in triplicate. The standard deviation for the NaOH concentration was <1%. Hydrochloric acid solutions were prepared by diluting appropriate quantities of concentrated HCl in MilliQ water. The exact concentration was determined by titrating against the titrated NaOH solution with 2 drops of phenolphthalein indicator; performed in triplicate. The standard deviation for the HCl concentration was <1%. KCl solutions were prepared by dissolving an appropriate amount of solid KCl in MilliQ water. $RECl_3$ solutions were prepared by dissolving appropriate quantities of the hydrated salts in the appropriate solvent.

Inductively Coupled Plasma Optical Emission Spectroscopy Measurements. Inductively coupled plasma optical emission spectroscopy (ICP-OES) measurements were performed using a Spectro Genesis ICP-OES spectrometer (SPECTRO Analytical Instruments GmbH, Kleve, Germany) equipped with an integrated three channel peristaltic pump and an ASX-260 auto-sampler (CETAC Technologies, Omaha, NE, USA). Samples solutions were delivered to the nebulizer using a Mod Lichte spray chamber and single-use PVC PT-2140PF tubing (Precision Glassblowing, Centennial, CO, USA). Each data acquisition was preceded by a 35 second rinse sequence at different pump speeds. Other relevant parameters were previously optimized to give the smallest RSD and are summarized in Table S1. Analytical plasma standard solutions were obtained from Alfa-Aesar (Specpure®, 1000 ppm of RE_2O_3 in 5% HNO_3). Calibrations were performed before every set of experiments using a range of 7 standardized

solutions (0-125 ppm). Calibration curves were confirmed to have $R^2 > 0.999$ for the selected elements. The following wavelengths (nm) were used for element quantifications and reviewed for absence of interferences: 333.749 (La), 364.540 (Dy), 430.358 (Nd) and were consistent with literature recommendations. Potential instrumental drift was monitored by continuously measuring Ar lines at 430.010 and 404.442 nm, and confirmed by analysis of a standard solution every 30 samples and verifying the metal concentration remained constant.

Supplementary Table 1. Instrumental and operation conditions for ICP-OES measurements.

**Instrument Conditions**	
Forward Power (W)	1350
Plasma gas flow rate (L min^{-1})	13.50
Auxiliary gas flow rate (L min^{-1})	1.00
Nebulizer Flow Rate (L min^{-1})	0.90
	La 333.749
Wavelengths (nm)	Nd 430.358
	Dy 364.540

HTE samples were prepared by diluting 100 μL of each filtrate with 10 mL 5% HNO_3 in MilliQ water. Separation experiment samples were prepared by digestion of 5-8 mg of analyte in 2 mL of freshly prepared 4:1 HNO_3 (Fisher, Certified ACS Plus grade)/H_2O_2 (Fisher, 30% solution in water) mixture at 100 °C for 5 minutes, followed by dilution with 10 mL MilliQ water to a concentration of *ca*. 5% HNO_3.
CAUTION: This digestion procedure is extremely oxidizing, releases NO_x gases, and should only be performed in a well-ventilated, hooded area while wearing appropriate personal protective equipment (PPE).

X-Ray Crystallography. X-ray intensity data were collected on a Bruker APEXII[1] CCD area detector or a Bruker APEXIII[2] D8QUEST CMOS area detector, both employing graphite-monochromated Mo-Kα radiation (λ = 0.71073 Å) at 100(1) K. Rotation frames were integrated using SAINT,[3] producing a listing of unaveraged F^2 and $\sigma(F^2)$ values which were then passed to the SHELXT program package[4] for further processing and structure solution. The intensity data were corrected for Lorentz and polarization effects and for absorption using SADABS or TWINABS.[5] Refinement was performed by full-matrix least squares based on F^2 using SHELXL2014.[4] All of the reflections were used during refinement. Non-hydrogen atoms were refined anisotropically and hydrogen atoms were refined using a riding model.

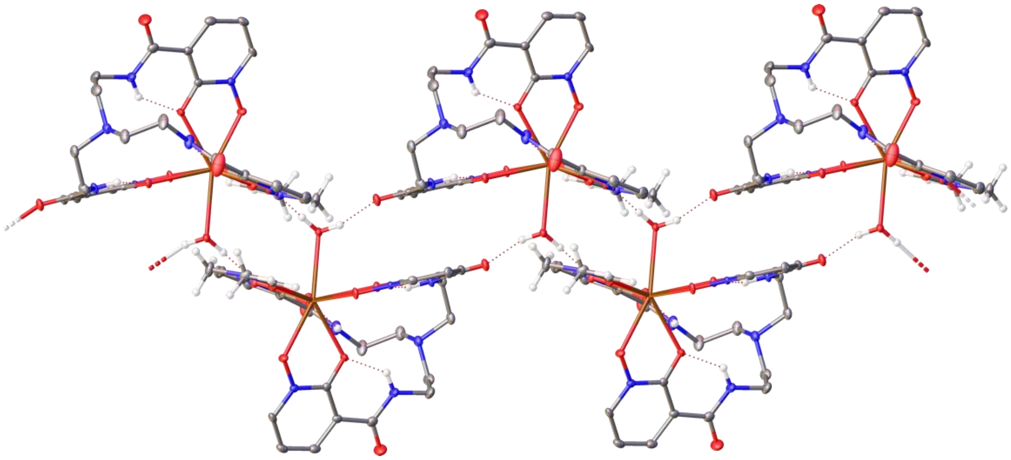

Supplementary Figure 1. Thermal ellipsoid plot of **1·La**(DMF)(H_2O) crystal packing. Dotted lines indicate intra- and inter-molecular hydrogen bonding to form a 1-D chain. All **1·RE**(DMF)(H_2O) (**RE** = La, Nd, Dy) complexes obtained exhibited the same H-bonding motif.

High Throughput Precipitation Screening. High throughput precipitation screening experiments were performed in 96-well reaction plates with a maximum volume of 2.00 mL per well. Experimental wells (EW) were loaded with 250 μL of the appropriate individual $RECl_3$ (66 mM, RE = La, Nd, Dy) solution followed by 600 μL of the appropriate ligand solution for a total volume of 850 μL ($[RE]_{initial}$ = 19.4 mM). Each set of experimental conditions were replicated in triplicate. Positive control (PC) wells were loaded with 250 μL of all three $RECl_3$ (RE = La, Nd, Dy) solutions and 100 μL solvent for a total volume of 850 μL and were placed after every 9 experimental wells. The well plate was covered with an adhesive aluminum foil cover to prevent solvent evaporation and cross-contamination between wells, then placed on an innova2180 platform shaker moving at 330 RPM for 24 hours. The reaction plate was centrifuged at 2000 RPM on a GeneVac EZ-2 Personal Evaporator for 1 hour. The supernatant was transferred to a 96-well filter plate with a maximum volume of 1.00 mL per well. Dynamic vacuum was applied, and the filtrate collected in a 96-well collection plate with a maximum volume of 2.00 mL per well. The filtrate was analyzed for metal content by ICP-OES. Reaction yields were calculated based on the ratio between RE concentration in the filtrates of the positive control and experimental wells according to Supplementary Equation 1.

$$\%\,Yield = \frac{[RE]_{PC} - [RE]_{EW}}{[RE]_{PC}} \times 100 \qquad \text{(Supplementary Equation 1)}$$

Supplementary Note: Yields from HTE screening are not calculated on the basis of collected mass. This method avoids the need to thoroughly dry the collected solid, transfer losses, and manually measuring individual masses in series, thereby saving time and improving precision between wells.

General Procedure for Separation of Rare Earth Mixtures. To a stirring solution of H_3**1**·TFA (1-2 equivalents) in the appropriate solvent (3.00 mL) was added a solution of **RE1**$Cl_3 \cdot nH_2O$ (0.083 mmol) and **RE2**$Cl_3 \cdot nH_2O$ (0.083 mmol) in the same solvent (1.25 mL). At the end of the reaction time (1-24 h), the mixture was filtered through a fine porosity sintered glass frit, and the solid washed twice with H_2O (0.5-1.0 mL), and once with acetone (0.5 mL, optional wash). The solid was dried on the frit, and the filtrate evaporated. The RE content of the solid and filtrate portions was analyzed by ICP-OES. All separation experiments were performed in triplicate and the results reported as the average of the three trials ± one standard deviation. Separation factors were calculated as the ratio of distribution coefficients between REs using data obtained from ICP-OES analysis[6] (*D* = distribution coefficient, *S* = separation factor, *EF* = enrichment factor, *n* = moles):

$$D_{RE} = \frac{[RE]_{solid}}{[RE]_{filtrate}} \qquad \text{(Supplementary Equation 2)}$$

$$SF_{RE2/RE1} = \frac{D_{RE2}}{D_{RE1}} = \frac{\frac{[RE2]_{solid}}{[RE2]_{filtrate}}}{\frac{[RE1]_{solid}}{[RE1]_{filtrate}}} = \frac{[RE2]_{solid}}{[RE2]_{filtrate}} \times \frac{[RE1]_{filtrate}}{[RE1]_{solid}} \qquad \text{(Supplementary Equation 3)}$$

$$EF_{filtrate} = \frac{n_{RE1,filtrate}}{n_{RE2,filtrate}} \qquad \text{(Supplementary Equation 4)}$$

$$EF_{solid} = \frac{n_{RE2,solid}}{n_{RE1,solid}} \qquad \text{(Supplementary Equation 5)}$$

Note: Enrichment factors were calculated in the precipitation-based separation method as a metric for the purity of the RE of interest within a single phase.

Procedure for Ligand Recovery. To solid **1·Dy** (20 mg, 0.02 mmol) was added minimal 12 M HCl (0.4 mL). Upon complete dissolution, EtOH (2.0 mL) was added with stirring. After 1 h, the mixture was centrifuged at 3000 RPM using a Thermo Scientific CL2 centrifuge for 30 minutes. The supernatant was decanted, and the solid suspended in fresh EtOH (1.0 mL). The mixture was centrifuged at 3000 RPM for an additional 30 minutes. The supernatant was decanted and the white solid dried in vacuo (H_3**1**·HCl, 10.6 mg, 84% yield, 12% residual **1·Dy** present). The combined supernatants were evaporated to yield off-white solid ($DyCl_3 \cdot nH_2O$, 4.9 mg, 86%). This procedure can be scaled up to 100 mg **1·Dy**, resulting in 88% recovery of H_3**1**·HCl (33% residual **1·Dy** present) and 80% recovery of $DyCl_3$.

Synthetic Details and Characterization

2-chloropyridine-3-carboxylic acid *N*-oxide (B)

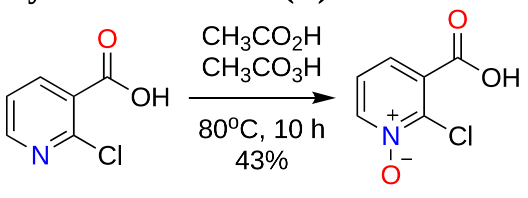

2-chloropyridine-3-carboxylic acid *N*-oxide (**B**) was synthesized according to previously reported literature procedures:[7]

To a stirring mixture of 2-chloronicotinic acid (12.00 g, 76.2 mmol) in glacial acetic acid (100 mL) was added peracetic acid (35%, 35 mL). The mixture was slowly heated to 80 °C, then allowed to stir at this temperature for 10 hours. The solution was allowed to cool to room temperature, then cooled in an ice bath. The resulting precipitate was filtered through a medium porosity fritted funnel, and washed with diethyl ether. The solid was dried under reduced pressure to afford 2-chloropyridine-3-carboxylic acid *N*-oxide (**A**) as a white solid (5.62 g, 43%). ^{1}H NMR (300 MHz, d_6-DMSO): δ = 14.14 (br s, 1H, CO_2H), 8.57 (d, 1H, $^3J_{HH}$ = 6 Hz, pyr-H), 7.67 (d, 1H, $^3J_{HH}$ = 6 Hz, pyr-H), 7.47 (t, 1H, $^3J_{HH}$ = 6 Hz, pyr-H). ^{13}C{^{1}H} NMR (400 MHz, d_6-DMSO): δ = 164.41, 141.76, 139.46, 131.92, 125.84, 124.07. ESI-MS m/z calc. for $C_6H_5ClNO_3$ $(M+H)^+$: 173.9958, found 173.9988.

1-hydroxy-2-oxo-1,2-dihydropyridine-3-carboxylic acid (C)

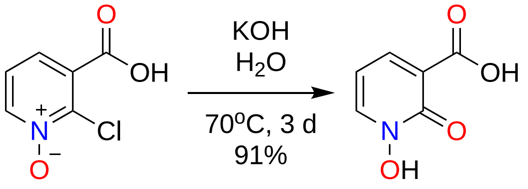

1-hydroxy-2-oxo-1,2-dihydropyridine-3-carboxylic acid was synthesized according to previously reported literature procedures:[7]

B (3.51 g, 20.2 mmol) was stirred in aqueous potassium hydroxide (10%, 60 mL) at 70 °C for 3 days. The solution was allowed to cool to room temperature, then cooled in an ice bath. The solution was then treated with concentrated hydrochloric acid until pH ~1 was reached. The crude product was filtered through a medium porosity fritted funnel, and washed with cold water to afford 1-hydroxy-2-oxo-1,2-dihydropyridine-3-carboxylic acid (**C**) as a white solid (2.86 g, 91%). ^{1}H NMR (300MHz, d_6-DMSO): δ = 13.69 (br s, 2H, OH), 8.44 (dd, 1H, $^3J_{HH}$ = 7 Hz, $^4J_{HH}$ = 1 Hz, pyr-H), 8.26 (dd, 1H, $^3J_{HH}$ = 7 Hz, $^4J_{HH}$ = 1 Hz, pyr-H), 6.43 (t, 1H, $^3J_{HH}$ = 7 Hz, pyr-H). ^{13}C{^{1}H} NMR (400 MHz, d_6-DMSO): δ = 164.53, 160.25, 142.26, 141.79, 117.12, 106.96. ESI-MS m/z calc. for $C_6H_4NO_4$ $(M-H)^-$: 154.0140, found 154.0157.

H_3tren-1,2,3-HOPO ($H_3$1·TFA)

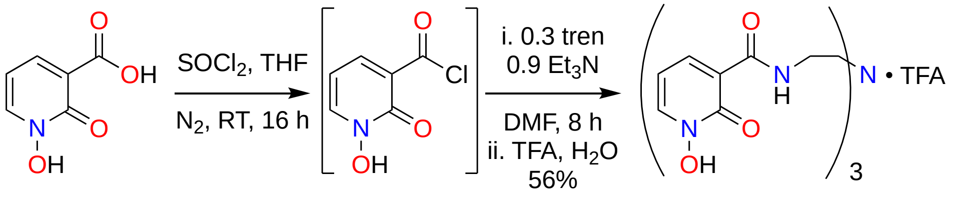

C H31•TFA

To a stirring suspension of **C** (4.00 g, 25.8 mmol) in dry THF (150 mL) under inert atmosphere was added thionyl chloride (9.4 mL, 129 mmol). After 16 h, volatiles were removed *in vacuo*, and the white residue suspended in dry DMF (50 mL). A solution of tris(2-aminoethyl)amine (tren, 1.13 g, 7.7 mmol) and Et_3N (2.35 g, 23.2 mmol) in dry DMF (10 mL) was added dropwise to the suspension with vigorous stirring. After 8 h at room temperature, the reaction mixture was slowly poured into 500 mL stirring Et_2O, forming solid. The solid was filtered and washed with Et_2O, then dissolved in water (25 mL) and trifluoroacetic acid (TFA, 1.5 mL). After 15-30 minutes, white solid precipitated. The solid was isolated by filtration and washed with minimal cold water yielding tris[(1-hydroxy-2-oxo-1,2-dihydropyridine-3-carboxamido)ethyl]amine (H_3tren-1,2,3-HOPO, H_3**1**·TFA) as an off-white solid (56%). Single crystals suitable for X-ray analysis were obtained via slow evaporation of a water solution of H_3**1**·TFA. Quantification by ^{1}H and ^{19}F NMR using an internal standard of *o*-$C_6H_4F_2$ has yielded a range of 0.77-1.93 TFA per ligand. ^{1}H NMR (500 MHz, D_2O): δ = 8.09 (d, $^3J_{HH}$ = 6 Hz, 3H, pyr-H), 7.83 (d, $^3J_{HH}$ = 7.5 Hz, 3H, pyr-H), 6.46 (t, $^3J_{HH}$ = 7 Hz, 3H, pyr-H), 3.91 (br t, $^3J_{HH}$ = 4.5 Hz, 6H, CH_2), 3.71 (br t, $^3J_{HH}$ = 5 Hz, 6H, CH_2). $^{13}C\{^1H\}$ NMR (500 MHz, D_2O with DMSO internal reference): δ = 168.32, 160.07, 143.12, 141.45, 120.71, 107.83, 55.62, 36.42. ESI-MS m/z calc. for $C_{24}H_{28}N_7O_9$ $(M+H)^+$: 558.1949, found 558.1931. Anal. calcd for $C_{28}H_{29}F_6N_7O_{13}$ [H_3tren-1,2,3-HOPO·2TFA]: C 42.81; H, 3.72; N, 12.48. Found: C 42.46; H 3.58; N 12.25.

La(tren-1,2,3-HOPO) (1·La)

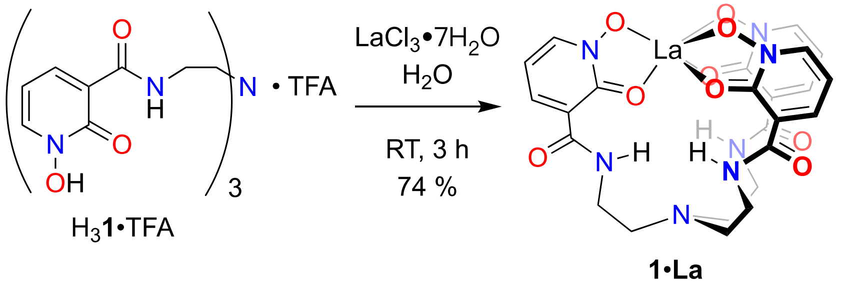

H31•TFA **1•La**

To a stirring solution of H_3**1**·TFA (0.18 mmol) in H_2O (2 mL) was added a solution of $LaCl_3·7H_2O$ (0.067 g, 0.18 mmol) in H_2O (1 mL) resulting in the immediate formation of a white solid. After 3 h, the reaction mixture was filtered and the solid washed with H_2O (3 × 1 mL). The solid was dried on the frit to afford **1·La** as a white solid (0.092 g, 74%). Single crystals suitable for X-ray analysis were obtained via vapor diffusion of H_2O into a solution of **1·La** in wet DMF. ^{1}H NMR (300 MHz, *d*$_6$-DMSO): δ = 9.80 (br s, 3H), 8.17 (br s, 3H), 7.95 (d, $^3J_{HH}$ = 6 Hz, 3H), 6.54 (t, $^3J_{HH}$ = 6 Hz, 3H), 3.20 (br s, 6H), 2.47 (br s, 6H, overlaps with solvent signal). $^{13}C\{^1H\}$ NMR (400 MHz, *d*$_6$-DMSO): δ = 163.53, 161.30, 139.15, 133.50, 115.97, 107.75, 55.51, 37.53. Anal. calcd for $C_{24}H_{34}LaN_7O_{14}$ [**1·La**$(OH_2)_4$]: C 37.66; H 4.21; N 12.81. Found: C 37.15; H 4.29; N 13.17.

Nd(tren-1,2,3-HOPO) (1·Nd)

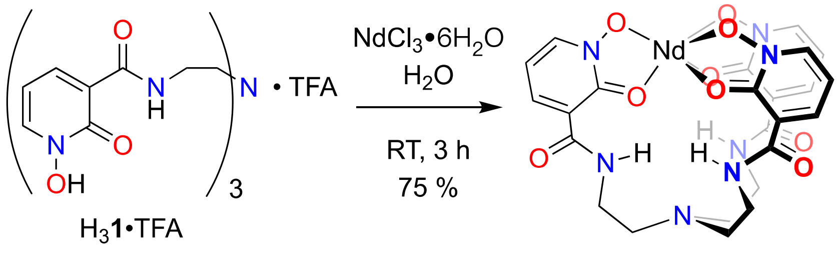

1·Nd

To a stirring solution of H_3**1**·TFA (0.18 mmol) in H_2O (2 mL) was added a solution of $NdCl_3·6H_2O$ (0.064 g, 0.18 mmol) in H_2O (1 mL) resulting in the immediate formation of white solid. After 3 h, the reaction mixture was filtered and the solid washed with H_2O (3 × 1 mL). The solid was dried on the frit to afford **1·Nd** as a pale blue-purple solid (0.094 g, 75%). Single crystals suitable for X-ray analysis were obtained via vapor diffusion of H_2O into a reaction of **1** in wet DMF layered on top of $NdCl_3·6H_2O$ in wet DMF. ^{1}H NMR (300 MHz, d_6-DMSO): δ = 17.53 (br s, 3H), 10.59 (br s, 3H), −0.13 (br s, 6H), −0.89 (br s, 6H), −2.89 (br s, 3H). Anal. calcd for $C_{24}H_{28}N_7NdO_{11}$ [**1·Nd** $(OH_2)_2$]: C 39.23; H 3.84; N 13.34. Found: C 39.42; H 4.05; N 13.14.

Dy(tren-1,2,3-HOPO) (1·Dy)

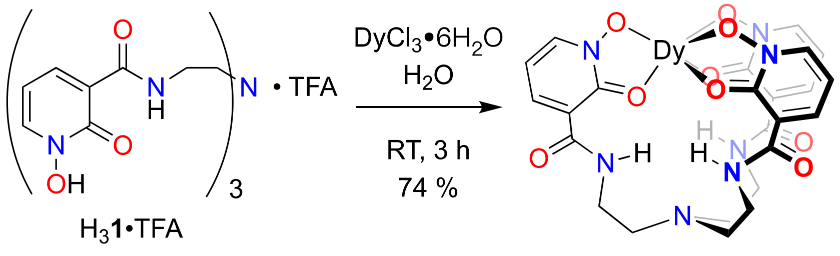

1·Dy

To a stirring solution of H_3**1**·TFA (0.18 mmol) in H_2O (2 mL) was added a solution of $DyCl_3·6H_2O$ (0.068 g, 0.18 mmol) in H_2O (1 mL) resulting in the immediate formation of white solid. After 3 h, the reaction mixture was filtered and the solid washed with H_2O (3 × 1 mL). The solid was dried on the frit to afford **1·Dy** as a white to off-white solid. The solid was dissolved in minimal DMF and precipitated via vapor diffusion of H_2O to afford **1·Dy**(DMF)(H_2O) as an off-white solid (0.095 g, 74%). Single crystals suitable for X-ray analysis were obtained via vapor diffusion of H_2O into a solution of **1·Dy** in wet DMF. ^{1}H NMR (300 MHz, DMSO-d_6): δ 73.86 (br s, 3H, N-H), 39.20 (br s, 3H, pyr-H), −61.32 (br s, 6H, CH_2), −62.55 (br s, 9H, CH_2, pyr-H), −249.70 (br s, 3H, pyr-H). Anal. calcd for $C_{24}H_{30}DyN_7O_{12}$ [**1·Dy**$(OH_2)_3$]: C 37.39; H 3.92; N 12.72. Found: C 37.36; H 3.91; N 12.41.

NMR Spectra

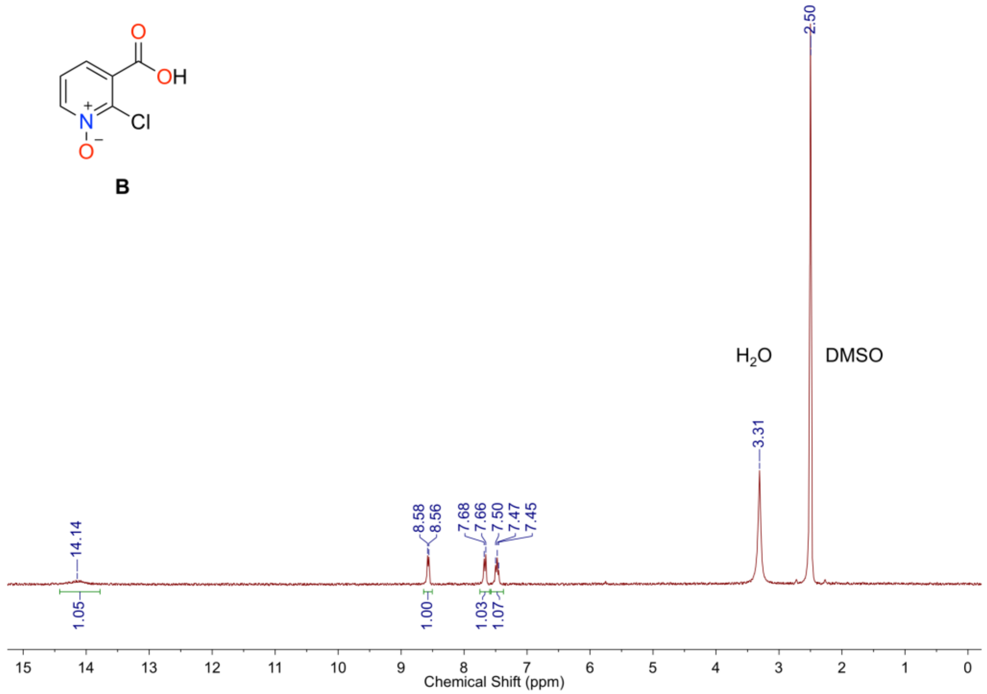

Supplementary Figure 2. ^{1}H NMR spectrum of **B** in DMSO-d_6.

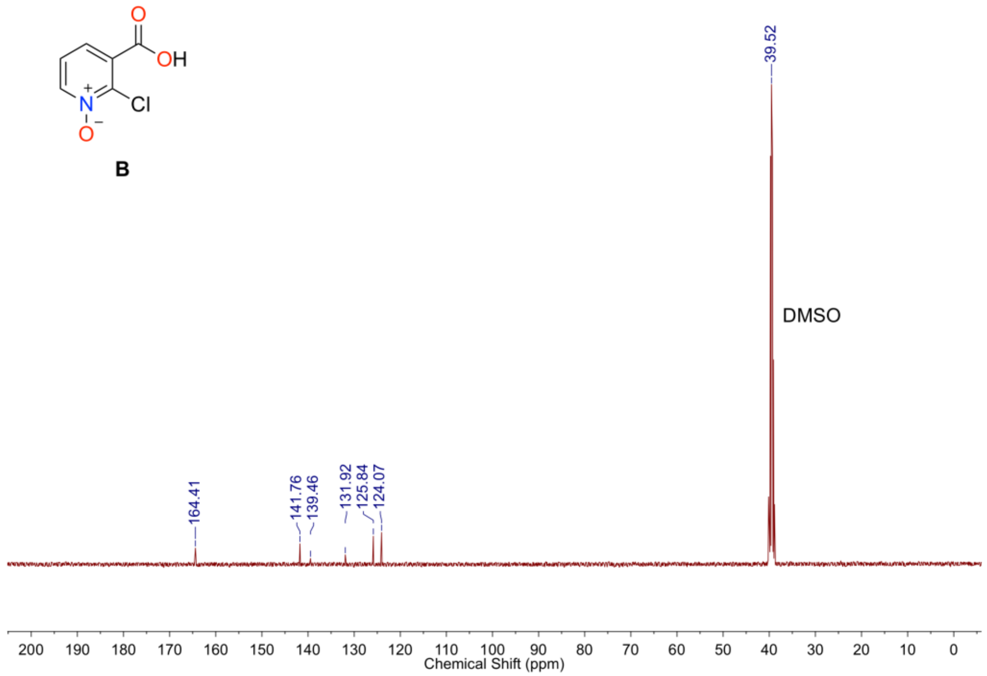

Supplementary Figure 3. $^{13}C\{^{1}H\}$ NMR spectrum of **B** in DMSO-d_6.

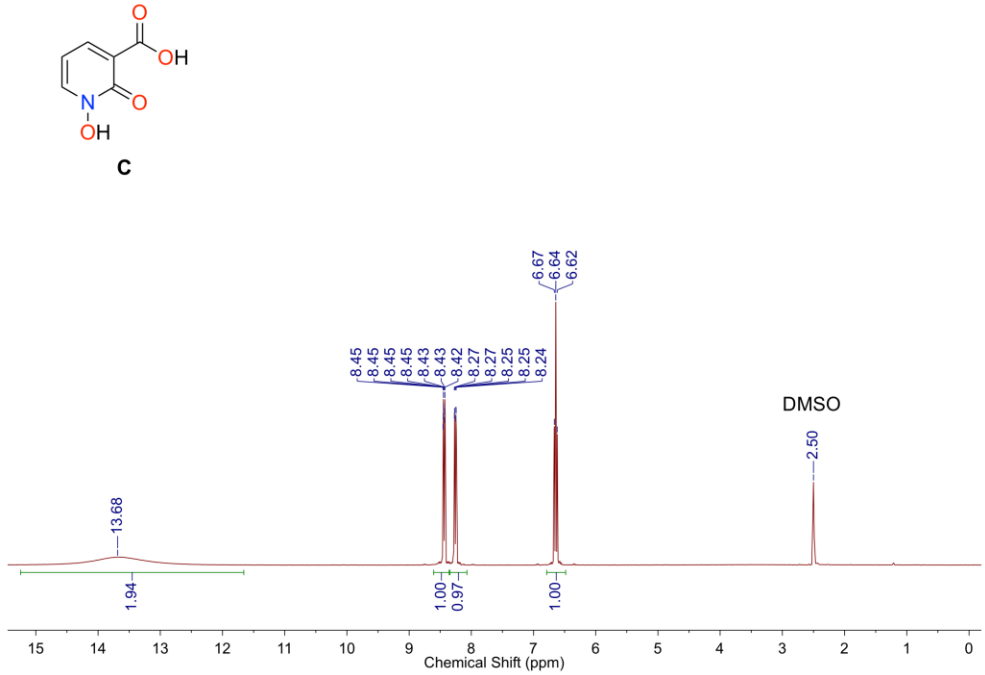

Supplementary Figure 4. ^{1}H NMR spectrum of **C** in DMSO-d_6.

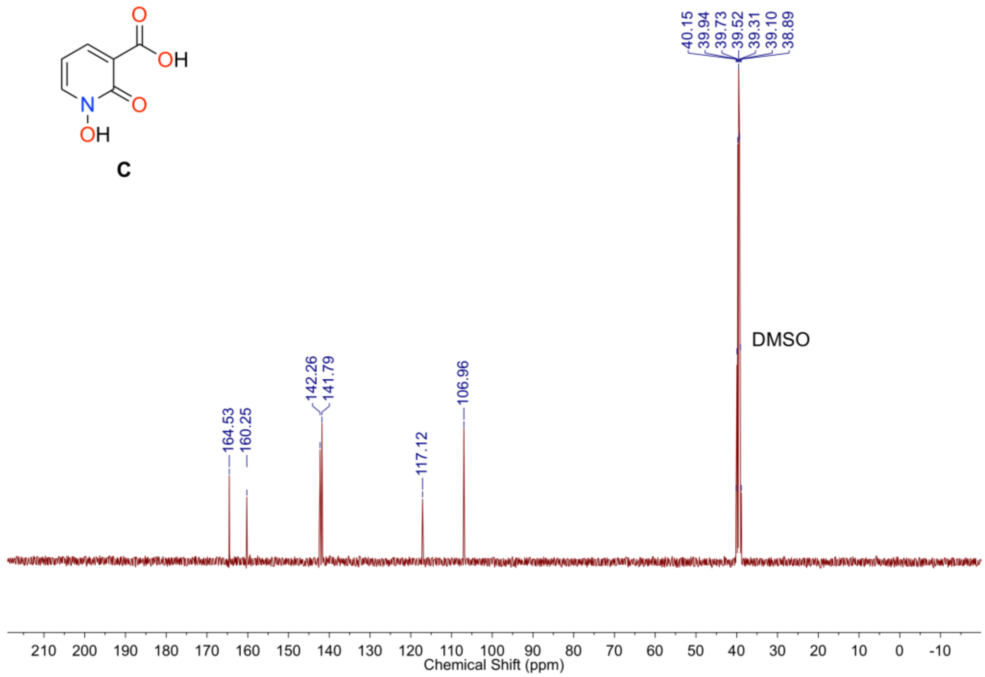

Supplementary Figure 5. ^{13}C{^{1}H} NMR spectrum of **C** in DMSO-d_6.

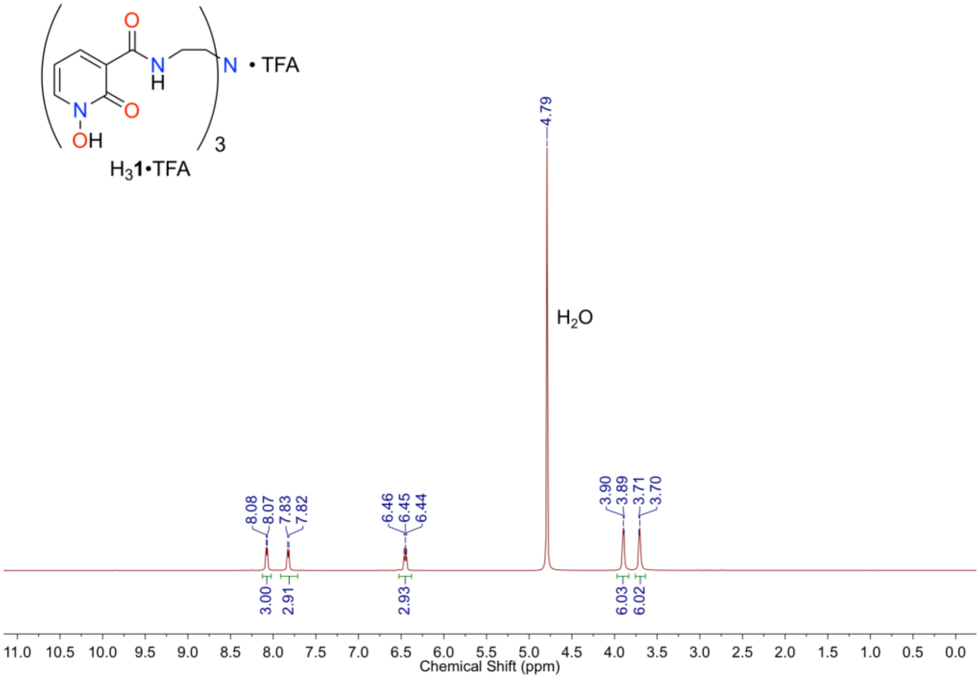

Supplementary Figure 6. ^{1}H NMR of H_3**1**·TFA in D_2O.

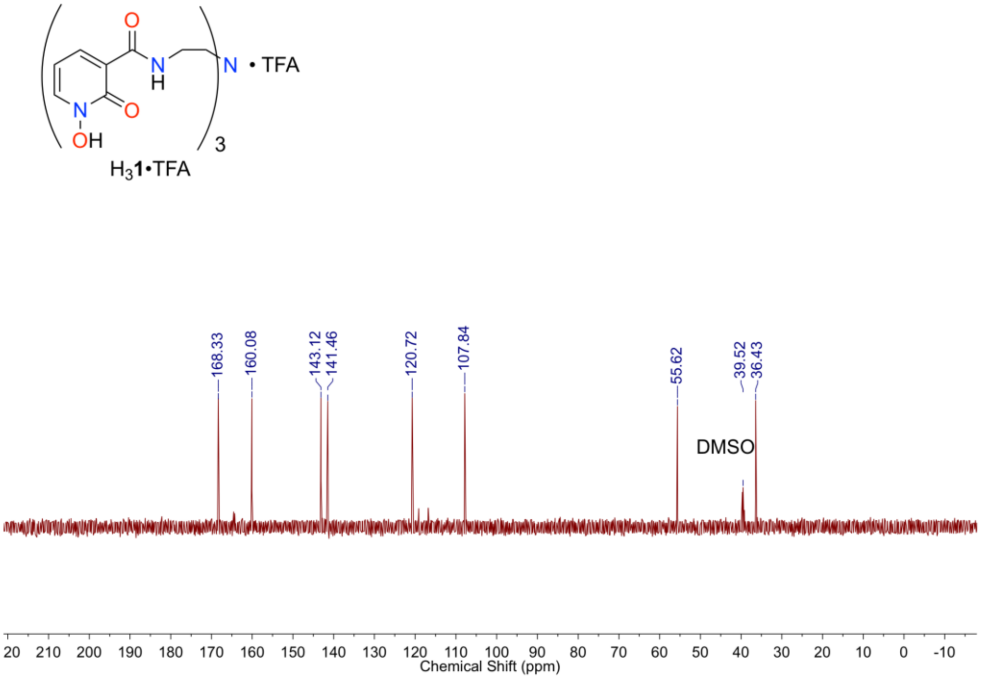

Supplementary Figure 7. $^{13}C\{^{1}H\}$ NMR of H_3**1**·TFA in D_2O with DMSO internal reference (39.52 ppm).

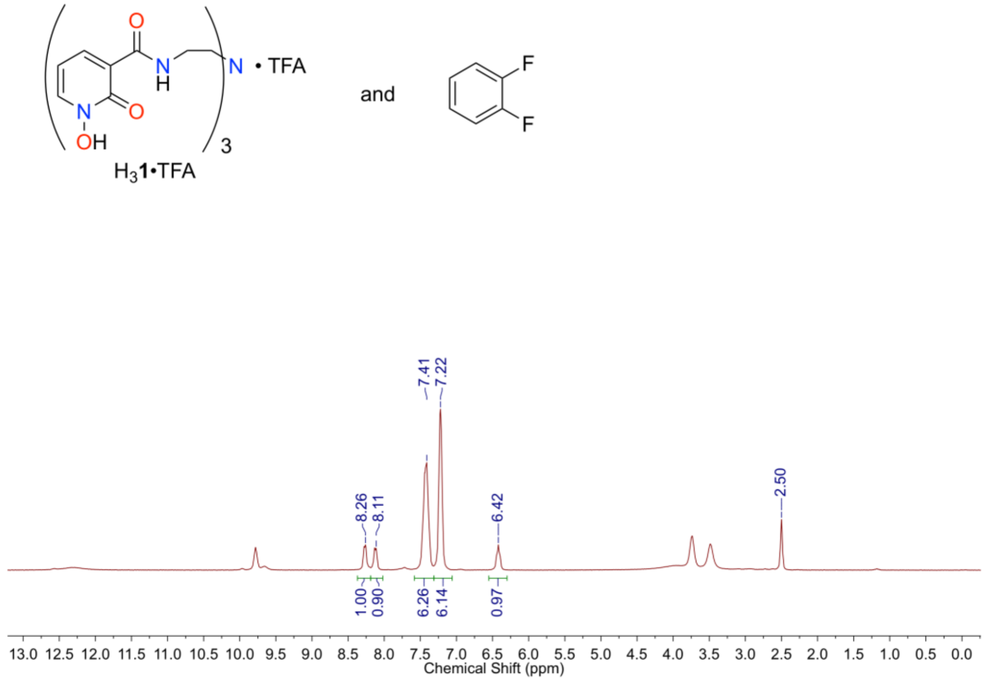

Supplementary Figure 8. ^{1}H NMR of H_3**1**·TFA with 1,2-difluorobenzene in DMSO-d_6.

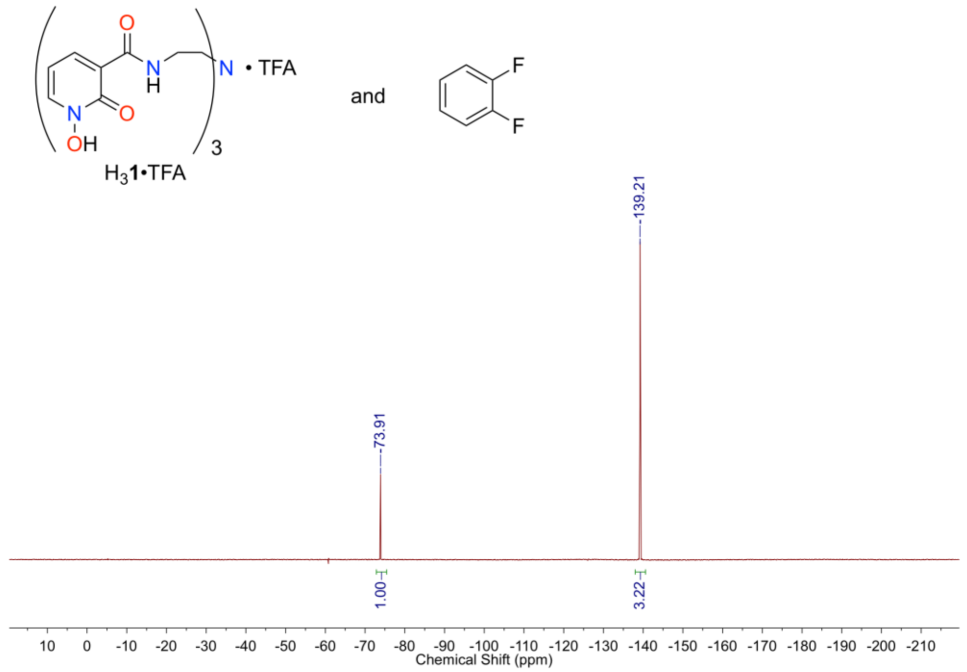

Supplementary Figure 9. ^{19}F NMR of H_3**1**·TFA with 1,2-difluorobenzene in DMSO-d_6.

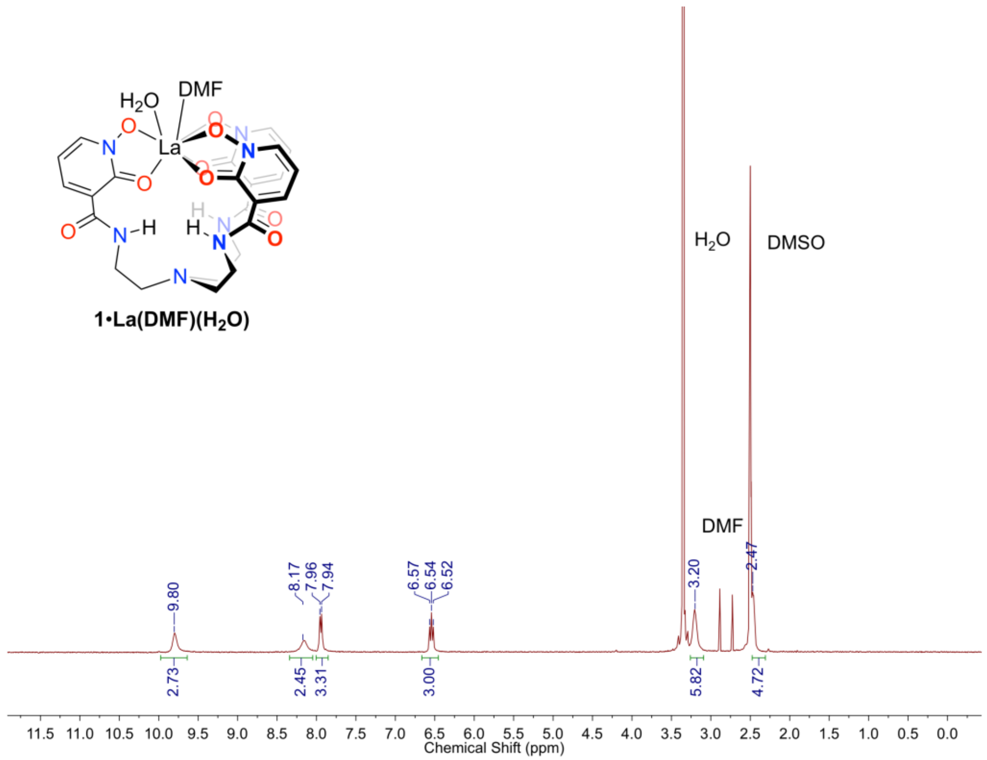

Supplementary Figure 10. ^{1}H NMR spectrum of **1·La**(DMF)(H_2O) in DMSO-d_6.

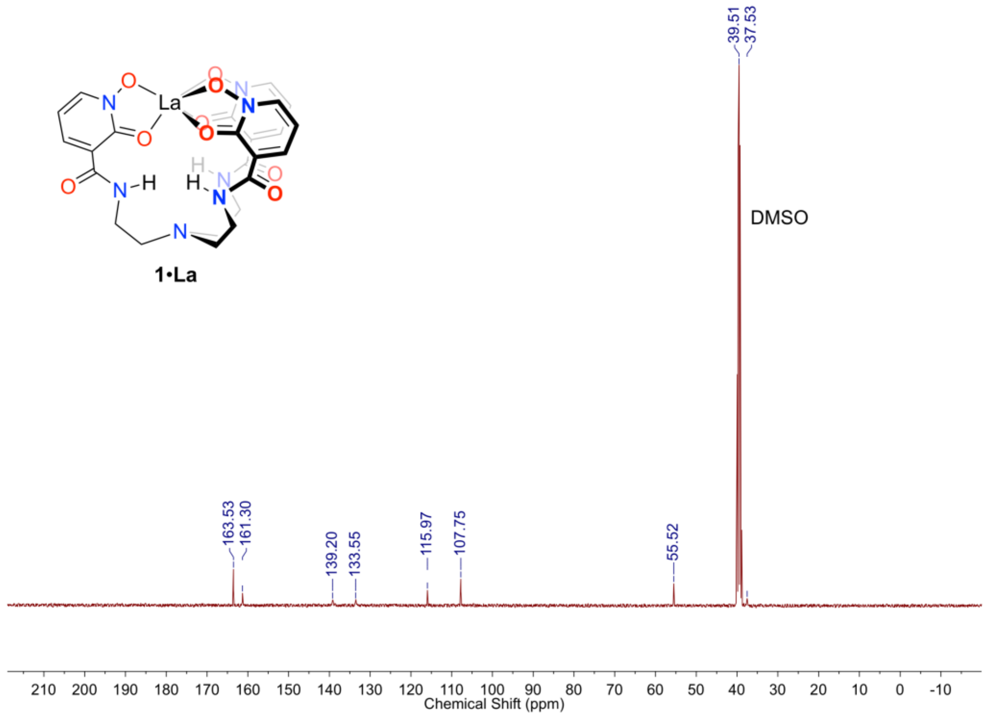

Supplementary Figure 11. $^{13}C\{^{1}H\}$ NMR of **1·La** in DMSO-d_6.

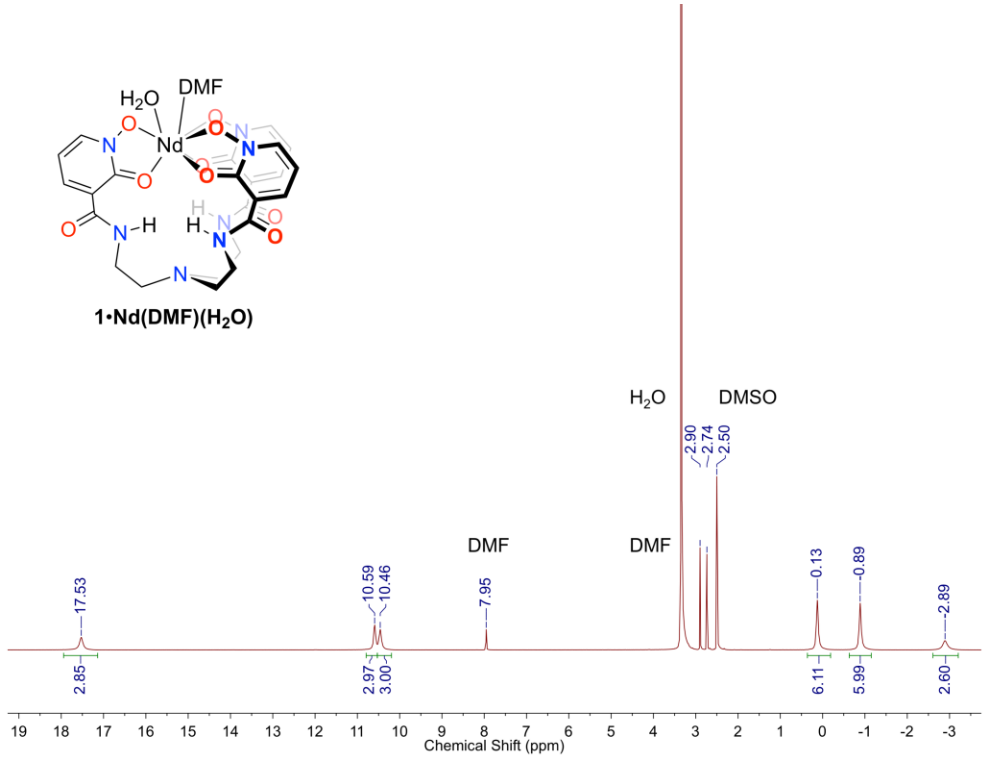

Supplementary Figure 12. ^{1}H NMR spectrum of **1**·**Nd**(DMF)(H_2O) in DMSO-d_6.

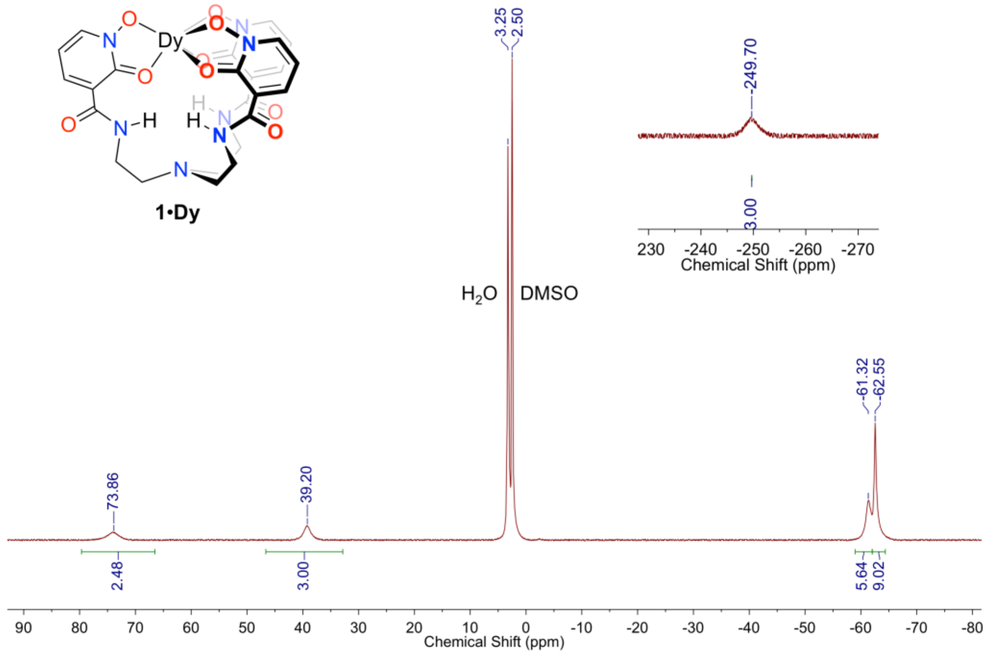

Supplementary Figure 13. ^{1}H NMR spectrum of **1**·**Dy** in DMSO-d_6.

FT-IR Spectra

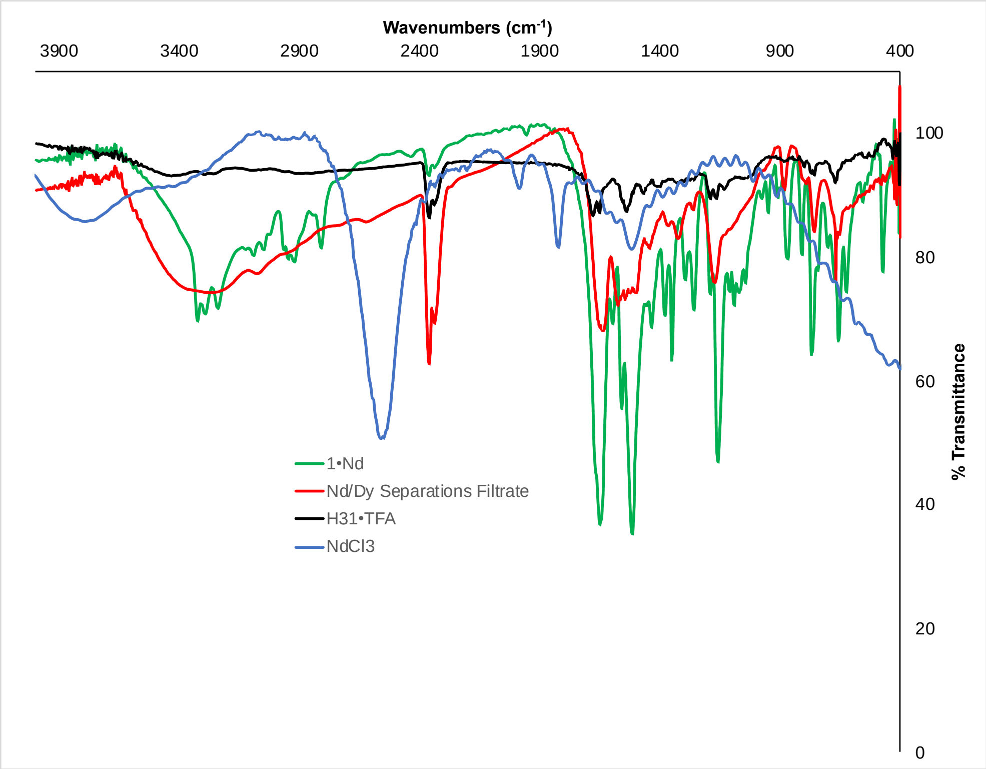

Supplementary Figure 14. FT-IR spectra of selected species. The filtrate portion of the Nd/Dy separations were not clearly one single species.

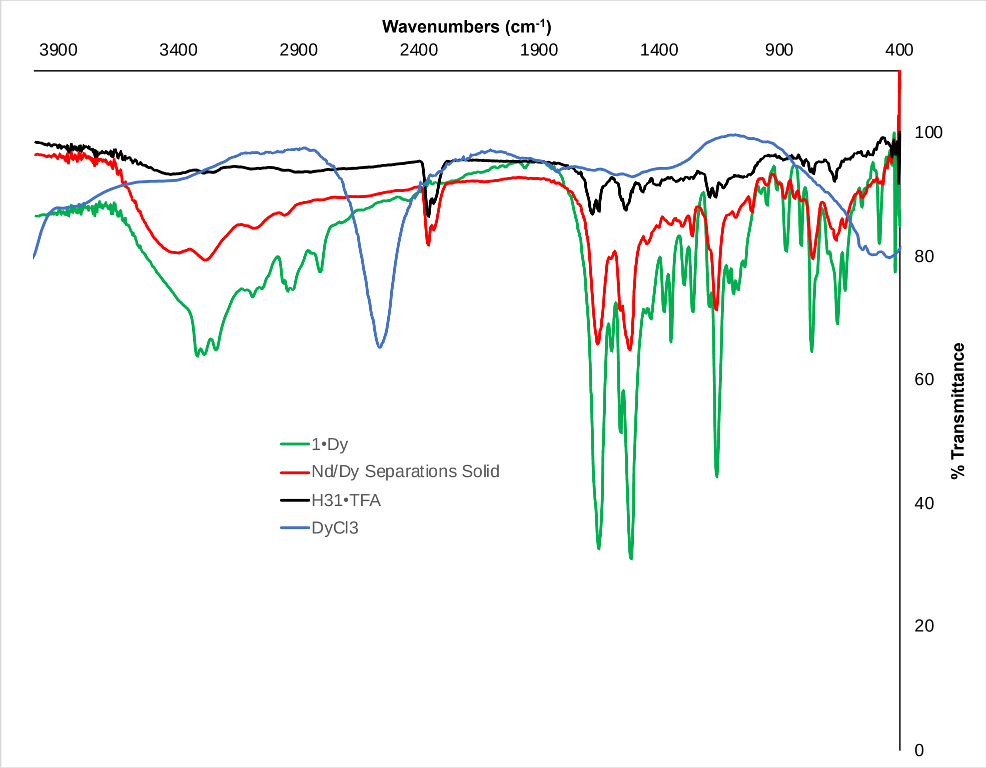

Supplementary Figure 15. FT-IR spectra of selected species. The solid portion of the Nd/Dy separations appears to be predominantly **1·Dy**.

Supplementary References

1. Bruker, *APEX 2 (v2014.11-0), Bruker AXS Inc., Madison, Wisconsin*, 2012.
2. Bruker, *APEX 3 (v2016.1-0), Bruker AXS Inc., Madison, Wisconsin*, 2015.
3. Bruker, *SAINT (v8.37a), Bruker AXS Inc., Madison, Wisconsin*, 2012.
4. G. M. Sheldrick, *Acta Crystallogr., Sect. A: Found. Adv.*, 2015, **71**, 3-8.
5. Bruker, *SADABS (v2014/5), Bruker AXS Inc., Madison, Wisconsin*, 2001.
6. T. Goto and M. Smutz, *J. Inorg. Nucl. Chem.*, 1965, **27**, 1369-1379.
7. D. Tetard and F. W. Lewis, GB Pat., WO 2016/079502 A1, 2016.
